# Na_V_1.9 Potentiates Oxidized Phospholipid-Induced TRP Responses Only under Inflammatory Conditions

**DOI:** 10.3389/fnmol.2018.00007

**Published:** 2018-01-23

**Authors:** Corinna Martin, Carolin Stoffer, Milad Mohammadi, Julian Hugo, Enrico Leipold, Beatrice Oehler, Heike L. Rittner, Robert Blum

**Affiliations:** ^1^Institute of Clinical Neurobiology, University Hospital Würzburg, University of Würzburg, Würzburg, Germany; ^2^Department of Anesthesiology, University Hospital Würzburg, Würzburg, Germany; ^3^Department of Biophysics, Center for Molecular Biomedicine, Friedrich Schiller University Jena and Jena University Hospital, Jena, Germany

**Keywords:** DRG neurons, inflammatory mediators, excitability, oxidized phospholipid, TRP ion channel, voltage-gated sodium channel, Na_V_1.9, calcium spikes

## Abstract

Oxidized phospholipids (OxPL) like oxidized 1-palmitoyl-2-arachidonoyl-sn-glycero-3-phosphocholine (OxPAPC) were recently identified as novel proalgesic targets in acute and chronic inflammatory pain. These endogenous chemical irritants are generated in inflamed tissue and mediate their pain-inducing function by activating the transient receptor potential channels TRPA1 and TRPV1 expressed in sensory neurons. Notably, prototypical therapeutics interfering with OxPL were shown to inhibit TRP channel activation and pain behavior. Here, we asked how OxPL excite primary sensory neurons of dorsal root ganglia (DRG neurons from mice of either sex). Acute stimulation of sensory neurons with the prototypical OxPL 1-palmitoyl-2-glutaryl-*sn*-glycero-3-phosphocholine (PGPC) evoked repetitive calcium spikes in small-diameter neurons. As Na_V_1.9, a voltage-gated sodium channel involved in nociceptor excitability, was previously shown to be essential for the generation of calcium spikes in motoneurons, we asked if this channel is also important for OxPL mediated calcium spike and action potential generation in nociceptors. In wild-type and Na_V_1.9-deficient neurons, the action potential firing rate and the calcium spike frequency to an acute PGPC stimulus was similar. When preincubated with inflammatory mediators, both, the action potential firing rate and the calcium spike frequency were markedly increased in response to an acute PGPC stimulus. However, this potentiating effect was completely lost in Na_V_1.9-deficient small-diameter neurons. After treatment with inflammatory mediators, the resting membrane potential of Na_V_1.9 KO neurons was slightly more negative than that of wild-type control neurons. This suggests that Na_V_1.9 channels are active under this condition and therefore increases the ease with which action potentials are elicited after OxPL stimulation. In summary, our data suggest that Na_V_1.9 has a switch function to potentiate the receptor potentials induced by OxPL under inflammatory conditions. Since human Na_V_1.9 has been shown to mediate painful and painless channelopathies, this study provides new insights into the mechanism by which Na_V_1.9 amplifies stimuli of endogenous irritants under inflammatory conditions.

## Introduction

Persistent or chronic pain syndromes can be initiated or maintained by a subpopulation of primary sensory neurons (Basbaum et al., 2009; Ji et al., 2016). Cell bodies of most nociceptive neurons are found in the dorsal root ganglia (DRG). The peripheral branch of DRG neurons senses pain stimuli and transmits sensory information to the spinal cord (Basbaum et al., 2009; Dubin and Patapoutian, 2010). Nociceptive DRG neurons mostly have small-diameter cell bodies and are primarily responsible for slow pain sensation via C- and Aδ-fibers (Dubin and Patapoutian, 2010; Denk et al., 2017). Persistent pain or inflammatory pain associated with certain diseases can result from changes in the signaling cascades responsible for nociception (Dubin and Patapoutian, 2010; Ji et al., 2014). These long-term changes in the signaling machinery can lead to prolonged and enhanced transmission of nociceptive signals (Basbaum et al., 2009; Bennett and Woods, 2014; Waxman and Zamponi, 2014; Habib et al., 2015). For instance, in inflammation the local chemical environment can induce peripheral sensitization of nociceptors and induces a higher spontaneous action potential firing rate, but also an increased responsiveness to endogenous or exogenous pain-inducing irritants (Basbaum et al., 2009; Dubin and Patapoutian, 2010). Targets of nociceptive stimuli are ion channels and receptors such as the transient receptor potential channel ankyrin-type 1 (TRPA1) and transient receptor potential channel vanilloid-type 1 (TRPV1), which are functionally expressed in small-sized C-fiber DRG neurons (Caterina et al., 1997, 2000; Tominaga et al., 1998; Story et al., 2003; Bautista et al., 2006; Julius, 2013).

Recently, we identified certain oxidized phospholipids (OxPL; Bochkov et al., 2010) as endogenous irritants of TRPA1 and TRPV1 ion channels in inflammatory and chronic pain (Oehler et al., 2017). OxPLs are a family of oxidation products and derive from endogenous phospholipids in biological membranes. To investigate the physiological and proalgesic function of OxPLs, the commercially available oxidized 1-palmitoyl-2-arachidonoyl-*sn*-glycero-3-phosphocholine (OxPAPC) was used for *in vitro* and *in vivo* experiments (Liu et al., 2016; Oehler et al., 2017). OxPAPC consists of a mixture of oxidized, chain-shortened phospholipids including POVPC [1-palmitoyl-2-(5-oxovaleroyl)-*sn*-glycero-3-phosphocholine], PGPC (1-palmitoyl-2-glutaryl-*sn*-glycero-3-phosphocholine), and oxygenated phospholipids like PEIPC [1-palmitoyl-2-(5,6)-epoxyisoprostaneE2-*sn*-glycero-3-phosphocholine; Bretscher et al., 2015; Oehler et al., 2017]. OxPL production is increased under oxidative stress and mediate inflammatory effects in certain disease conditions, for instance in atherosclerosis plaque formation (Furnkranz et al., 2005). Recently we showed that prototypical therapeutics, a monoclonal antibody against OxPL (EO6; Palinski et al., 1996; Binder et al., 2003), as well as a mimetic peptide deduced from apolipoprotein A1 (D4F; Van Lenten et al., 2008), inhibit the function of OxPL on TRPA1 and TRPV1 *in vitro* and *in vivo* and reduce pain behavior *in vivo* (Liu et al., 2016; Oehler et al., 2017). However, OxPL are also present in non-inflamed tissue and OxPL-induced inward currents are relatively small and long-lasting (Oehler et al., 2017), thus raising the question how OxPL signals are transmitted. Furthermore, OxPL act acutely and are rather reactive and instable irritants (Oehler et al., 2017). OxPAPC-induced hyperalgesia develops over hours (Oehler et al., 2017), indicating that acute effects cannot fully explain its proalgesic potency.

Transduction and transmission of pain signals after injury or inflammation depends in part on increased excitability of primary sensory neurons. Nociceptive neurons express multiple subtypes of voltage-gated sodium channels, of which subtype Na_V_1.9 possess unique features such as subthreshold activity, cell-autonomous activity and slow gating kinetics that may influence increased responsiveness to endogenous pronociceptive irritants such as OxPL (Wetzel et al., 2013; Bennett and Woods, 2014; Dib-Hajj et al., 2015).

In this study, we asked how signals from defined OxPLs are transmitted to excite DRG neurons. We found that the action potential firing rate and the calcium spike frequency in response to an acute OxPL stimulus are markedly increased when small DRG neurons were pre-treated with inflammatory mediators. This inflammatory mediator-dependent potentiation of the stimulus is fully lost in Na_V_1.9-deficient small-diameter DRG neurons.

## Materials and methods

### Reagents and chemicals

The following reagents were used: allyl-isothiocyanate, (AITC, Sigma), 4-(3-chloro-2-pyridinyl)-N-[4-(1,1-dimethylethyl)phenyl]-1-piperazinecarboxamide (BCTC, Sigma), bradykinin (BK), capsaicin (Caps, Alomone Labs), ω-conotoxin GIVA (Ascent scientific), forskolin (Fors, Abcam), HC-030031 (Sigma), Histamine (His, Sigma), nifedipine (Nif, Sigma), oxidized 1-palmitoyl-2-arachidonoyl-*sn*-glycero-3-phosphocholine (OxPAPC, Hycultech), PGPC (Avanti Polar Lipids), prostaglandin E2 (PGE_2_, Sigma), tetrodotoxin (TTX, Ascent scientific). Dimethyl sulfoxide, chloroform or aqueous physiological solutions served as solvents.

### Animals

C57BL/6J wild type mice and Na_V_1.9 KO (SCN11a^−/−^) mice (Östman et al., 2008), backcrossed to C57BL/6J for at least 9 generations, were bred in Institute of Clinical Neurobiology, University Hospital of Würzburg, Germany. All animals were kept under a nonsterile housing environment in accordance with the local Animal Care and Use Guidelines. All experiments and study protocols were performed in accordance with the European Union guidelines, and were approved by our institutional Animal Care and Utilization Committee and the Regierung von Unterfranken, Würzburg, Germany.

### Cell culture

HEK293 cells (ACC305; Leibniz Institute DSMZ, Braunschweig, Germany) were maintained in DMEM, 4.5 g glucose, 1 mM L-glutamine, 10% FCS, 1% penicillin/streptomycin. Cells were transfected with Lipofectamine 2000 (Invitrogen) and a vector expressing TRPA1-3C (Macpherson et al., 2007). Cells were prepared for ratiometric calcium imaging at day 2 post transfection as described below.

### Preparation of OxPLs

OxPLs were solved in chloroform and stored in glass vials. Before storing, the chloroform was evaporated with nitrogen to prevent further oxidation. Then OxPL were stored at −20°C. For experiments, OxPL were freshly solved in the appropriate extracellular solution for calcium imaging or electrophysiological recording.

### Culture of primary sensory neurons

Primary DRG cultures were prepared from 6 to 12 weeks old wild-type and Na_v_1.9 null mutant mice of either sex. Mice were euthanized by CO_2_ asphyxiation and cervical dislocation. DRG neurons were removed from all parts of the spinal cord and treated with Liberase Blendzyme 4 (TH; 5U/500 μl, Sigma) and Liberase Blendzyme 3 (TM; 5U/500 μl, Sigma) (Dib-Hajj et al., 2009). DRGs were grown in DMEM/F12 high glucose containing glutamax, 10% FCS, 1% PenStrep (all Life Technologies) and 100 ng/ml nerve growth factor (NGF, Sigma) at 37°C, 5% CO_2_ atmosphere. DRG neurons were plated at a density of 7 × 10^3^ cells on 10 mm glass cover slips (Marienfeld, Lauda-Königshofen, Germany) coated with poly-L-lysine (20 μg/ml). Patch clamp recording and calcium imaging measurements were performed within 24–36 h after plating the cells. Fixation and staining of the cells was performed at day *in vitro* (DIV) 2–3. At DIV 2 and 3, the cell culture contained neurons with pronounced axonal structures and cells with hallmarks of glial cells (Supplementary Figure 1).

### Immunocytochemistry

For immunocytochemistry, cells on cover glasses were fixed (4% paraformaldehyde/PBS,15 min) and then permeabilised and blocked for 1 h with blocking solution (PBS, 10% horse serum, 0.1% Tween 20, 0.3% Triton X-100). Primary antibodies were incubated in blocking solution for 3 h at room temperature. The following antibodies were used: mouse anti-TUJ1 (Neuromics, MO15013, RRID:AB_1624212, 0.5 μg/ml, TRPA1 (Novusbio, NB110-40763SS, RRID:AB_715124, 1/1000), goat anti-TRPV1 (Santa Cruz, sc-12503, RRID:AB_2209139, 1/400) and rabbit anti-Na_V_1.9 (T71n, 1/500; Subramanian et al., 2012). Both anti-TRP antibodies were tested to be specific for recombinant TRPA1 and TRPV1 expressed in HEK293 cells. Secondary antibodies were used at a concentration of 0.5 μg/ml: Alexa-488 conjugated goat anti-mouse IgG (H+L) (Life technologies), donkey anti-rabbit IgG-Cy3, and donkey anti-mouse Cy5 (Jackson ImmunoResearch) in blocking solution for 1 h. Nuclei were labeled with DAPI (400 ng/ml) in PBS. Cells were mounted in Aqua-Poly/Mount (Polysciences). Confocal image acquisition was performed with an IX81 microscope combined with an Olympus FV1000 confocal laser scanning system, a FVD10 SPD spectral detector and diode lasers of 405, 473, 559, and 635 nm using an Olympus UPLSAPO40x objective (oil, numerical aperture, NA, 1.35). Pinhole setting represented one airy disk. Twelve-bit z-stack images were adjusted in brightness and contrast using ImageJ software, RRID: SCR_003070 (Schneider et al., 2012). Final figure preparation was carried out using Adobe Photoshop CS5. Images are shown as RGB images.

### Calcium imaging

For ratiometric single cell calcium analysis, DRG neurons were labeled with 3 μM Fura-2/AM in 20% Pluronic in DMSO for 30 min at 37°C in imaging solution (in mM): 134 NaCl, 6 KCl, 1 MgCl_2_, 1 CaCl_2_, 10 HEPES, 5.5 glucose, pH 7.4 adjusted with NaOH. All measurements were performed at room temperature using a Nikon TE2000-E microscope. Fura-2 AM was excited for 60–90 ms with 340/380 nm with a Lambda DG4/17 wavelength switch (Sutter Instruments). Time-lapse image series were acquired at intervals of 2 s with a cooled EMCCD Andor iXon camera (Andor Technology) controlled by NIS Elements Software (Nikon) with a 10x CFI S-Fluor objective (N.A. 0.5; Nikon). Image series were analyzed with ImageJ. The following agonists were used at indicated concentration: OxPAPC (10 μM), PGPC (10 μM), AITC (10 μM), capsaicin (1 μM), and KCl (50 mM). For inhibition experiments, cells were pretreated for 10 min with BCTC (1 μM) and HC-030031 (10 μM). For calcium imaging under inflammatory conditions, cover slips with DRG neurons were pre-incubated in DMEM containing an inflammatory milieu (inflammatory soup: 5 nM bradykinin, 100 nM histamine, 50 nM PGE_2_), bradykinin (200 nM), or forskolin (10 μM), at 37°C for 30 min before calcium imaging experiments. The cells of the control group were incubated in DMEM alone.

For fast calcium imaging, the high-affinity calcium indicator Oregon Green 488 BAPTA-1, AM, was prepared as 5 mM stock solution of 20% Pluronic F-127 (both Life technologies) solved in DMSO. For dye loading of cells, Oregon Green 488 BAPTA-1 AM, was solved in calcium imaging buffer (119 mM NaCl, 4.5 mM KCl, 1 mM MgCl_2_, 2 mM CaCl_2_, 1.2 mM NaH_2_PO_4_, 26 mM NaHCO_3_, 10 mM Glucose, 10 mM HEPES) and primary DRG neurons were incubated for 15 min in a standard cell culture incubator. Subsequently the cover glasses were transferred to the imaging setup and imaged under continuous perfusion (~5x chamber volume per min). For steady flow of the solution, the Minipuls 3 Peristaltic Pump (Gilson) was used. For PGPC stimulation we used a perfusion chamber made of aluminum with anodized surface, or ceramic. The imaging setup consisted of a BX51WI upright microscope (Olympus) equipped with a 20× water-immersion objective (Olympus UMPLanFL N; NA 0.5), a coolLED epifluorescent light source for 470 nm (Visitron Systems), equipped with a Rolera XR Mono fast 1394 CCD camera (Qimaging). Image sequences were acquired using the streaming software Streampix 4.0 (NorPix). Images were captured at a speed of 5 Hz.

For investigation of PGPC-induced calcium spike inhibition (**Figure 3**), DRG neurons (DIV 1) were stimulated with PGPC (30 μM) for 5 s, 0.2 bar, at RT with the help of a pneumatic drug ejection system (PDES-02DX, NPI electronic) and a glass pipette. Cells were kept under continuous perfusion (5x chamber volume exchange per min using a Minipuls 3 Peristaltic Pump; Gilson). After PGPC stimulation, either TTX (500 nM), nifedipine (5 μM), ω-conotoxin (CTX; GVIA; 30 nM) or a combination of HC-030331 (10 μM) and BCTC (1 μM) were applied with the help of the perfusion system. Inhibitors were washed out for at least 20 min before cells were again stimulated with PGPC in order to verify the recovery from activity inhibition. Vehicle applications served as control.

For analysis, fluorescent intensities in defined regions of interest (ROIs) were obtained using the ImageJ software. Results were transferred and analyzed with Origin Pro 9.0 (OriginLab Corporation).

### Whole cell patch clamp recordings

For electrophysiological experiments with DRG neurons, an extracellular solution containing 120 mM NaCl, 3 mM KCl, 2.5 mM CaCl_2_, 1 mM MgCl_2_, 30 mM HEPES, and 15 mM glucose (pH 7.4 with NaOH) was used (Leipold et al., 2013). The pipette solution contained 125 mM KCl, 8 mM NaCl, 1 mM CaCl_2_, 1 mM MgCl_2_, 0.4 mM Na_2_-GTP, 4 mM Mg-ATP, 10 mM EGTA, and 10 mM HEPES (pH 7.3 with KOH, ~110 nM free Ca^2+^). Current clamp recordings were acquired in the *whole-cell* configuration of the patch clamp method from isolated DRG neurons with an electrical capacitance of <30 pF, to delimit the recordings to small-diameter neurons only (Leipold et al., 2013). The resting membrane potential (RMP) was determined by zero current injection directly after establishing the *whole-cell* configuration. Voltage clamp recordings were performed at a holding potential of −60 mV which closely resembles the native resting membrane voltage of small diameter DRG neurons. The exchange of bath solutions occurred via a perfusion pipette, which was located near the cell during the measurement. Patch-pipettes with 2.5–5 MΩ resistances were pulled from borosilicate glass (GB 150-8P, Science Products) with a P-97 micropipette puller (Sutter Instruments). Data were acquired using a HEKA EPC-10 USB patch-clamp amplifier controlled by the PatchMaster software (HEKA Electronic). Raw data were continuously sampled at a frequency of 5 kHz and filtered at 2.9 kHz. Agonists were applied with a second glass pipette controlled by a pneumatic drug ejection system directly before current injection (0.5 s, 0.2 bar; PDES-02DX, NPI electronic).

### Experimental design and statistics

For each experiment, genotype and sample size are described in the corresponding figure legends. Replicates were performed with primary neurons prepared from different animals of either sex. The exact number of cells per experiment and the number of animals used to prepare these cells are given in the figure legends. Data are presented as mean ± S.E.M. To compare two dependent, normally distributed samples, a paired *t*-test was performed. Statistical significance in multiple measurements of two variables was computed by one-way ANOVA repeated measurements (RM) and post Holm-Sidak test, as we compared selected, but independent pairs of means. All acquired data were included to the statistical analysis and no data were excluded by an outlier test. Differences were considered significant when *p* ≤ 0.05 (^*^), *p* ≤ 0.01 (^**^), or *p* ≤ 0.001 (^***^). Exact *p*-values are listed in the figure legends or in Tables 1–3. Statistical analysis was performed with OriginPro 9.0 (OriginLab Corporation, Northampton, MA, USA).

**Table 1:** Statistics of either the basal and extracellular, PGPC or OxPAPC induced AP frequency.

**Basal and Extracellular solution**	***t*-value**	***p*-value**
0 pA	–	–
20 pA	*t*_(14)_ = 1	0.36
40 pA	*t*_(14)_ = 1	0.36
60 pA	*t*_(14)_ = 0.68	0.52
80 pA	*t*_(14)_ = −1.16	0.29
100 pA	*t*_(14)_ = 0	0
120 pA	*t*_(14)_ = −0.88	0.41
140 pA	*t*_(14)_ = −1.11	0.30784
**Basal and OxPAPC**	***t*****-value**	***p*****-value**
0 pA	–	–
20 pA	*t*_(12)_ = 2.24	0.076
40 pA	*t*_(12)_ = 3.22	0.024^*^
60 pA	*t*_(12)_ = 6.43	0.0014^**^
80 pA	*t*_(12)_ = 4.51	0.01^*^
100 pA	*t*_(12)_ = 2.98	0.031^*^
120 pA	*t*_(12)_ = 2.40	0.062
140 pA	*t*_(12)_ = 2.44	0.059
**Basal and PGPC**	***t*****-value**	***p*****-value**
0 pA	*t*_(14)_ = 1.93	0.1
20 pA	*t*_(14)_ = 2.87	0.0283^*^
40 pA	*t*_(14)_ = 3.93	0.0077^**^
60 pA	*t*_(14)_ = 5.18	0.0021^**^
80 pA	*t*_(14)_ = 4.41	0.0045^**^
100 pA	*t*_(14)_ = 3.16	0.02^*^
120 pA	*t*_(14)_ = 3.06	0.022^*^
140 pA	*t*_(14)_ = 1.99	0.094

**Table 2:** Statistics of the difference of basal and PGPC respectively OxPAPC induced AP frequency [ΔAP_(stim−basal)_].

	**PGPC**	***F*-value**	***p*-value**	**OxPAPC**	***F*-value**	***p*-value**
0 pA	PGPC + HC-030031	*F*_(2, 15)_ = 2.27	0.090^*^	OxPAPC + HC-030031	*F*_(2, 12)_ = 2.69	1
	PGPC + BCTC		0.11	OxPAPC + BCTC		1
	HC-030031+ BCTC		1	HC-030031 + BCTC		1
20 pA	PGPC + HC-030032	*F*_(2, 15)_ = 4.74	0.022^*^	OxPAPC + HC-030031	*F*_(2, 12)_ = 5.04	0.073
	PGPC + BCTC		0.025^*^	OxPAPC + BCTC		0.098
	HC-030031+ BCTC		0.91	HC-030031 + BCTC		1
40 pA	PGPC + HC-030033	*F*_(2, 15)_ = 7.98	0.0038^**^	OxPAPC + HC-030031	*F*_(2, 12)_ = 17.86	0.025^*^
	PGPC + BCTC		0.0081^**^	OxPAPC + BCTC		0.027^*^
	HC-030031+ BCTC		0.88	HC-030031 + BCTC		0.87
60 pA	PGPC + HC-030034	*F*_(2, 15)_ = 10.73	0.0014^**^	OxPAPC + HC-030031	*F*_(2, 12)_ = 17.86	0.00041^***^
	PGPC + BCTC		0.0029^**^	OxPAPC + BCTC		0.0009^***^
	HC-030031+ BCTC		0.58	HC-030031 + BCTC		0.41
80 pA	PGPC + HC-030035	*F*_(2, 15)_ = 8.78	0.0032^**^	OxPAPC + HC-030031	*F*_(2, 12)_ = 7.86	0.0037^**^
	PGPC + BCTC		0.0051^**^	OxPAPC + BCTC		0.029^*^
	HC-030031+ BCTC		0.67	HC-030031 + BCTC		0.21
100 pA	PGPC + HC-030036	*F*_(2, 15)_ = 3.06	0.034^*^	OxPAPC + HC-030031	*F*_(2, 12)_ = 3.56	0.029^*^
	PGPC + BCTC		0.14	OxPAPC + BCTC		0.13
	HC-030031+ BCTC		0.41	HC-030031 + BCTC		0.35
120 pA	PGPC + HC-030037	*F*_(2, 15)_ = 2.36	0.049^*^	OxPAPC + HC-030031	*F*_(2, 12)_ = 1.17	0.16
	PGPC + BCTC		0.22	OxPAPC + BCTC		0.45
	HC-030031+ BCTC		0.41	HC-030031 + BCTC		0.49
140 pA	PGPC + HC-030037	*F*_(2, 15)_ = 1.47	0.12	OxPAPC + HC-030031	*F*_(2, 12)_ = 1.86	0.1
	PGPC + BCTC		0.23	OxPAPC + BCTC		0.2
	HC-030031+ BCTC		0.72	HC-030031 + BCTC		0.64

**Table 3:** Statistics of the AP frequency of wt and Na_V_1.9 KO DRG neurons stimulated either with PGPC or PGPC and inflammatory soup (IS).

**Steady state**	**Steady state**	**0 pA**	**20 pA**	**40 pA**	**60 pA**
		***F*_(3, 27)_ = 3.73**	***F*_(3, 27)_ = 6.14**	***F*_(3, 27)_ = 7.67**	***F*_(3, 27)_ = 9.66**
Basal wt	PGPC wt	0.012^*^	0.0011^**^	0.0018^**^	0.00083^***^
Basal wt	Basal Na_V_1.9 KO	1	1	0.92	0.69
Basal Na_V_1.9 KO	PGPC Na_V_1.9 KO	0.012^*^	0.012^*^	0.0037^**^	0.001^**^
PGPC wt	PGPC Na_V_1.9 KO	1	0.33	0.69	0.63
**IS**	**IS**	***F***_(3, 20)_ = **1.23**	***F***_(3, 20)_ = **3.62**	***F***_(3, 20)_ = **4.07**	***F***_(3, 20)_ = **3.79**
Basal wt	PGPC wt	0.24	0.032^*^	0.012^*^	0.020^*^
Basal wt	Basal Na_V_1.9 KO	0.52	0.53	0.81	0.58
Basal Na_V_1.9 KO	PGPC Na_V_1.9 KO	0.78	0.95	0.94	0.94
PGPC wt	PGPC Na_V_1.9 KO	0.15	0.013^*^	0.011^*^	0.018^*^
**Steady state**	**Steady state**	**80 pA**	**100 pA**	**120 pA**	
		***F***_(3, 27)_ = **8.11**	***F***_(3, 27)_ = **2.61**	***F***_(3, 27)_ = **1.20**	
Basal wt	PGPC wt	0.002^**^	0.042^*^	0.17	
Basal wt	Basal Na_V_1.9 KO	0.77	0.93	0.76	
Basal Na_V_1.9 KO	PGPC Na_V_1.9 KO	0.002^**^	0.092	0.29	
PGPC wt	PGPC Na_V_1.9 KO	0.77	0.64	0.54	
**IS**	**IS**	***F***_(3, 20)_ = **2.89**	***F***_(3, 20)_ = **2.55**	***F***_(3, 20)_ = **3.065**	
Basal wt	PGPC wt	0.047^*^	0.072	0.052	
Basal wt	Basal Na_V_1.9 KO	0.56	0.56	0.5	
Basal Na_V_1.9 KO	PGPC Na_V_1.9 KO	0.83	0.96	0.93	
PGPC wt	PGPC Na_V_1.9 KO	0.028^*^	0.031^*^	0.02^*^	

## Results

### PGPC can serve as a prototypical OxPL agonist

To establish a more defined neuronal stimulation with an oxidized phospholipid, we first tested whether PGPC can serve as a prototypical OxPL compound. PGPC offers several advantages over OxPAPC mixtures: (1) It has a lower chemical reactivity and is a truncated end product during PAPC oxidation (Frühwirth et al., 2007; Bochkov et al., 2010); (2) it is not electrophilic, but activates typical physiological OxPL responses via TRPA1 (Oehler et al., 2017); (3) it shows an overall high biological activity (Bochkov et al., 2010; Stemmer and Hermetter, 2012), (4) provides more reliable stimulus conditions (see later), and (5) it is commercially available as a purified substance.

**Figure 1:**
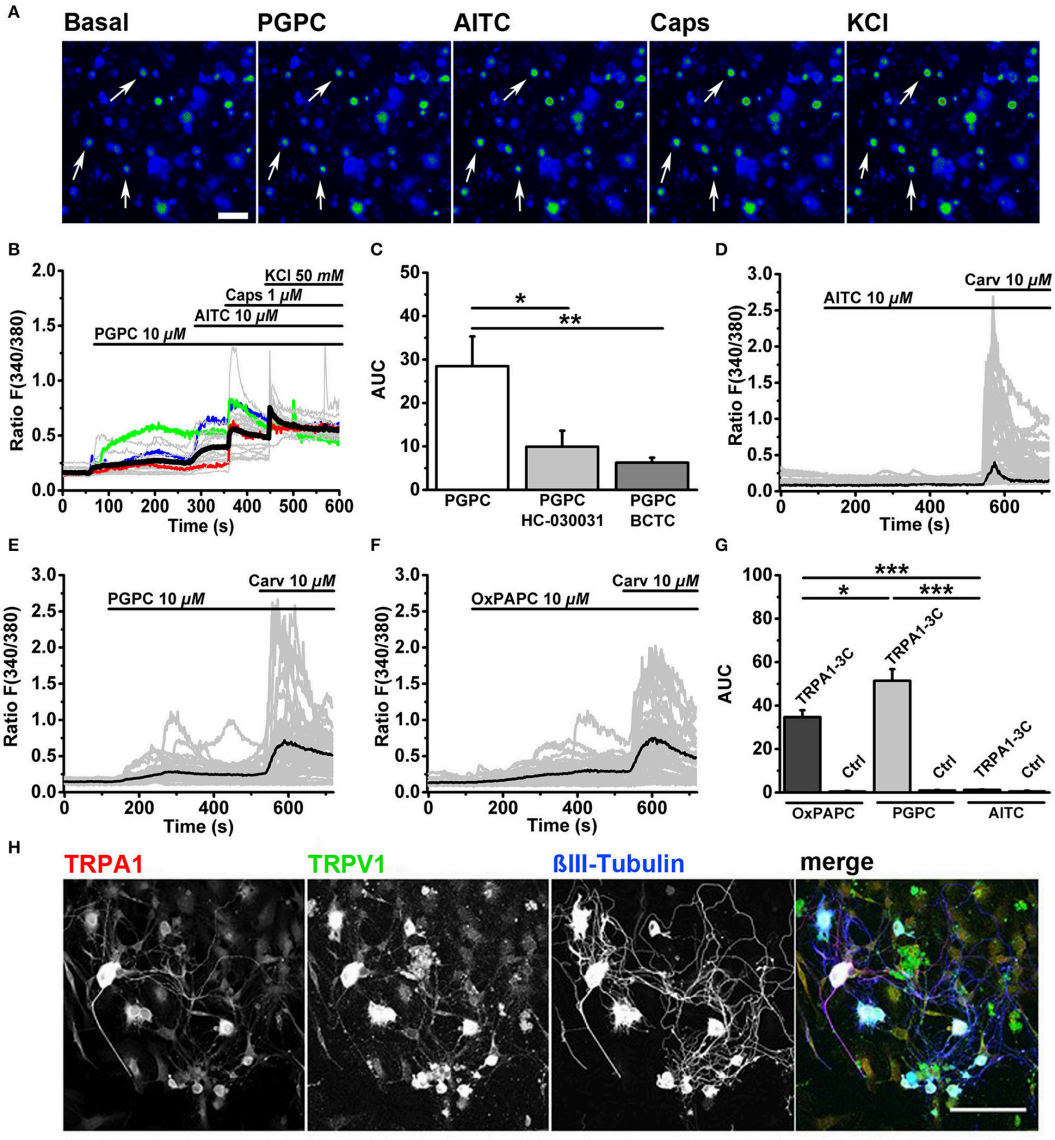
OxPL-evoked calcium influx is TRPA1- and TRPV1-dependent. **(A)** Pseudo colored images represent a calcium imaging series of adult murine DRG neurons loaded with Fura-2-AM. Calcium responses evoked by consecutive addition of PGPC (10 μM), AITC (10 μM), capsaicin (Caps, 1 μM), and KCl (50 mM) are displayed in comparison to the start situation (Basal; scale bar = 100 μm). Arrows indicate PGPC-responding neurons. **(B)** Relative changes in (Ca^2+^)_i_ evoked by consecutive application of PGPC (10 μM), AITC (10 μM), Caps (1 μM), and KCl (50 mM) in DRG neurons. Gray traces indicate calcium responses of individual cells, ratio *F*_(340/380)_. Black line: Mean of 50 cells. Colored lines (red, blue, green) represent three kinds of PGPC affected neurons. **(C)** Relative calcium responses of neurons activated by PGPC in the absence (PGPC) or after preincubation with antagonists specific for TRPA1 (PGPC + HC-030031) or TRPV1 (PGPC + BCTC) expressed as area under the curve, AUC, [*n* = 4-6 of 3 mice; mean ± SEM; one-way ANOVA Holm-Sidak; *F*_(2, 14)_ = 5.83; ^*^p < 0.028, ^**^p < 0.008]. **(D)** Relative changes in (Ca^2+^)_i_ of HEK293 expressing TRPA1-C3 induced by application of either OxPAPC (10 μM; mean of *n* = 50 cells), **(E)** PGPC (10 μM; mean of *n* = 50 cells), or **(F)** AITC (10 μM; mean of *n* = 50 cells) with consecutive addition of carvacrol (10 μM). Gray traces represent calcium responses [*F*_(340/380)_] of individual cells, black traces indicate means. **(G)** The relative calcium responses of HEK293 cells transfected with TRPA1-3C (TRPA1-3C) and untransfected control cells (Ctrl), stimulated with either OxPAPC, PGPC or AITC expressed as area under the curve [Ctrl; *n* = 5–6 of 3 cultures; data points are presented as means ± SEM; one-way ANOVA Holm-Sidak; *F*_(2, 16)_ = 42.33; ^*^p ≤ 0.05, ^***^p < 0.001]. **(H)** Indirect immunofluorescent staining of 48 h old primary DRG cultures from adult wt mice against TRPA1 (red), TRPV1 (green) and βIII-Tubulin (blue; scale bar = 100 μm).

First we performed ratiometric calcium imaging at a speed of 0.5 Hz and stimulated DRG neurons in an imaging chamber with consecutive stimuli, as described recently (Oehler et al., 2017). PGPC evoked a sustained increase in cytosolic calcium in DRG neurons (Figures 1A–C). After PGPC, DRG neurons were stimulated with the exogenous irritants AITC, a mustard oil compound and agonist of TRPA1, capsaicin, a hot chili pepper compound and agonist of TRPV1, and KCl to identify neurons. As shown in Figure 1B, stimulation of neurons with PGPC produced rather heterogeneous calcium responses. Most neurons showed only brief increases in cytosolic calcium concentrations upon PGPC application (e.g., red and blue traces in Figure 1B), some neurons responded with a strong increase of intracellular calcium (e.g., green trace in Figure 1B). Similar to earlier experiments with the OxPL mixture OxPAPC (Oehler et al., 2017), PGPC responses were reduced when cells were stimulated in presence of the TRPA1 antagonist HC-030031, and BCTC, an inhibitor of TRPV1 responses. In accordance with earlier findings (Oehler et al., 2017), no cellular response was observed when only bath solution was applied (*n* = 84 cells, two culture preparations; AUC: mean value = 0.05 ± 0.038 S.E.M).

### PGPC stimulates recombinant TRPA1-3C channels

Our study (Oehler et al., 2017) as well as another study (Liu et al., 2016) showed that OxPL induced calcium signals via TRPA1 were dramatically reduced or even absent when critical intracellular reactive cysteine and lysine residues in the N-terminus of the protein were mutated. In both studies, OxPAPC was used as agonist. This indicated that OxPAPC requires cysteine and/or lysine residues for covalent activation of TRPA1. However, OxPAPC preparations also contain PGPC (Oehler et al., 2017), which also triggers calcium responses in DRG neurons (Figure 1B), but is for chemical reasons unable to react with cysteine residues. Thus, OxPL-mediated activation of TRPA1 is unlikely to depend solely on covalent TRPA1 activation mechanisms. Therefore, we expressed the TRPA1 mutant (hTRPA1-3C), with three critical cysteines replaced by serine (C621S/C641S/C665S) in HEK293 cells. AITC, which acts through these cysteine residues, failed to activate hTRPA1-3C (Figures 1D,G), while carvacrol, a TRPA1 agonist, which acts through a non-electrophilic binding site on TRPA1, was still able to activate the channel (Figures 1D–G). The non-electrophilic PGPC also activated this mutant (Figures 1E,G), and this OxPAPC preparation could also activate TRPA1-3C (Figures 1F,G). This is in line with the concept that the OxPAPC mixture, which contains also PGPC (Bretscher et al., 2015; Oehler et al., 2017), can act also in a non-electrophilic manner on TRPA1.

To verify whether the OxPL target receptors TRPA1 and TRPV1 are present in our adult DRG cell preparations, we performed immunofluorescence labeling of both TRP ion channels. Immunocytochemistry was performed on 48 h old DRG neurons *in vitro*. Cells were counterstained with an antibody against βIII-Tubulin in order to identify neurons. TRPA1 was abundant in the soma (Figure 1H, red) and most TRPA1 positive cells also showed anti-TRPV1 immunoreactivity (Figure 1H, green). Both ion channels were present in large and small DRG neurons and were even visible in neurites. Both antibodies showed a tendency to label non-neuronal cells, which also appear in the primary DRG culture. These cells show hallmarks of glial cells (Supplementary Figure 1) and do not respond to capsaicin or AITC. We therefore assume that the label of the glial cells is due to a weak unspecific cross-reactivity of the corresponding antibodies.

### PGPC induces repetitive calcium spikes in small-diameter DRG neurons

**Figure 2:**
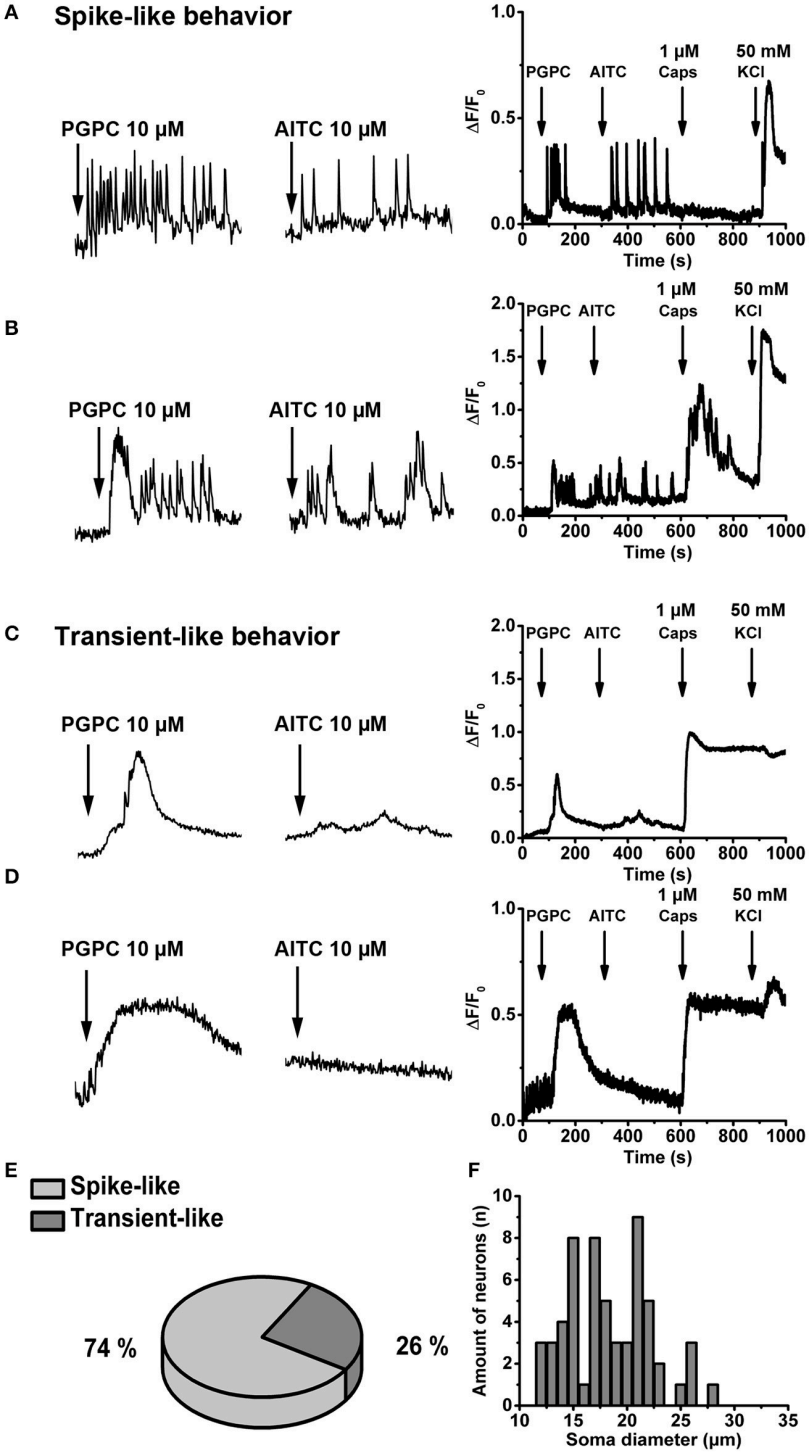
OxPL evoke repetitive, spike-like calcium transients in DRG neurons**. (A,B)** *Left* Representative examples of calcium spikes obtained from two wild-type DRG neurons stimulated with PGPC following AITC. Neurons were loaded with Oregon Green BAPTA-1, AM, and time-lapse imaging was performed under continuous perfusion with calcium imaging buffer using an imaging rate of 5 Hz. *Right* Relative changes in (Ca^2+^)_i_ evoked by consecutive application of PGPC (10 μM), AITC (10 μM), Caps (1 μM), and KCl (50 mM) expressed as ΔF/F_0_ as a function of time. **(C,D)** Identical experiments as in **A,B** showing two representative cells which respond to PGPC stimulation with slow calcium transients. **(E)** Distribution of calcium response behaviors of PGPC responsive DRG neurons. **(F)** Distribution of the soma size of PGPC positive neurons (in **E,F**: *n* = 59 cells).

The response intensity to a PGPC stimulus in individual neurons was quite diverse (Figure 1B), suggesting that neurons might differ in their response behavior. To address this hypothesis, primary DRGs were cultured for 1 day, loaded with the calcium indicator Oregon Green BAPTA-1, AM, a high-affinity calcium indicator useful to investigate calcium transients and spikes in neurons (Grienberger and Konnerth, 2012). Calcium imaging was performed at a higher resolution (see Material and Methods) and under continuous perfusion (~5x chamber volume per min) and images were acquired at 5 Hz, meaning at 10-fold higher frequency compared to Figure 1B. With this imaging condition, PGPC induced spike-like calcium responses in DRG neurons became visible (Figures 2A,B; Supplementary Video 1). Some neurons also showed this spike-like response behavior on an AITC stimulus (Figures 2A,B). However, the AITC-induced calcium spikes occurred after stimulation with PGPC. As PGPC-induced ion currents are quite long-lasting (Oehler et al., 2017), it might be that the PGPC inward currents contribute to this AITC response behavior in this experiment. The subsequent capsaicin stimulus to activate TRPV1 elicited a response in 16% of the PGPC responsive cells (*n* = 255 cells, 12 coverslips from 3 mice), while 26% showed a large, but slow calcium transient (examples in Figures 2C–E). Notably, 74% of all PGPC-positive neurons showed spike-like calcium transients upon PGPC stimulation (Figure 2E). Based on imaging data, we measured the soma size of the PGPC-responsive primary neurons. The mean soma diameter was 18.5 ± 4 μm (*n* = 60 cells, 12 coverslips of 3 mice, Figure 2F). The experiments revealed that PGPC does not solely induce a longer lasting calcium influx event, but can also generate faster spike-like calcium transients, which last 40–100 s.

**Figure 3:**
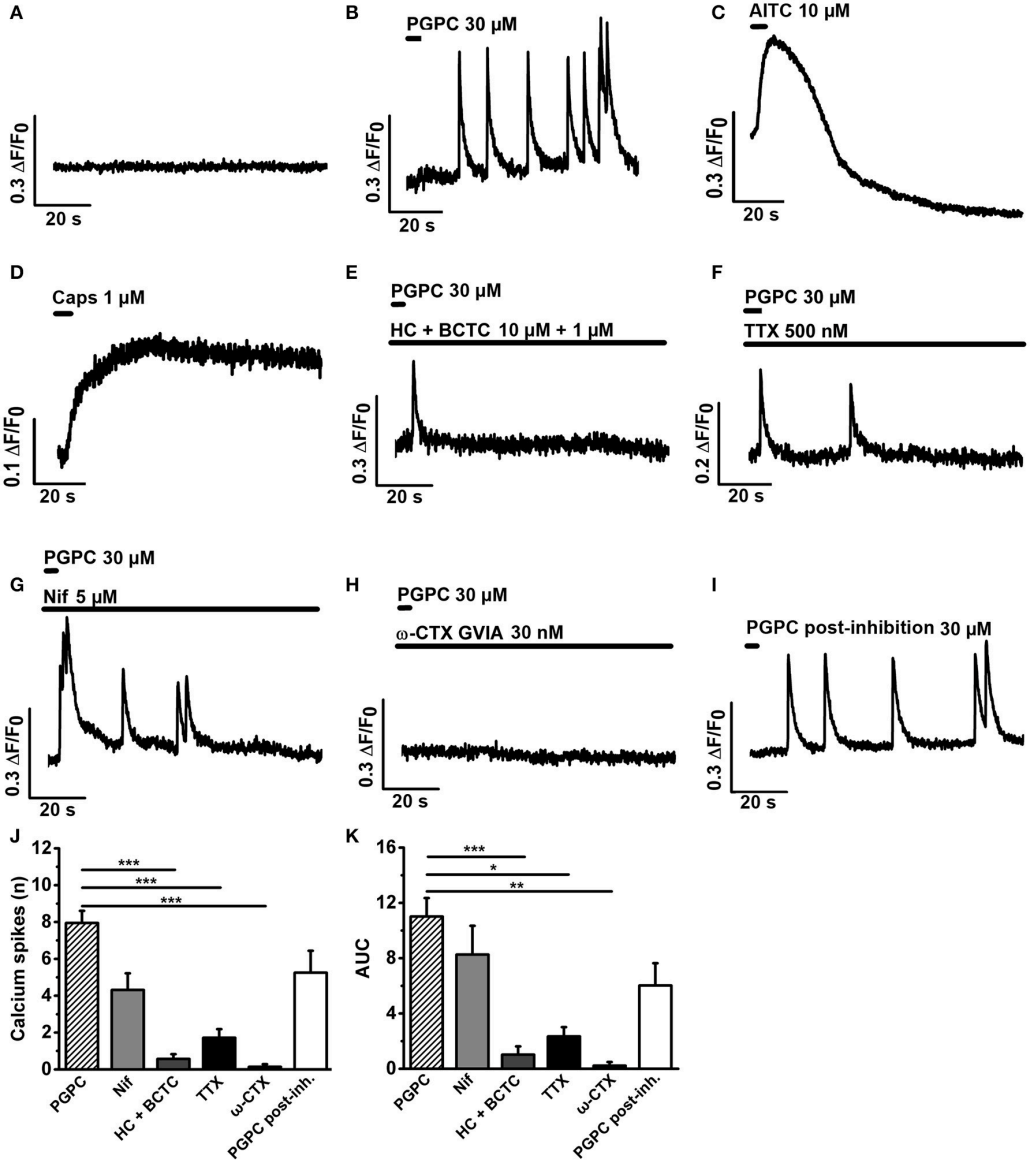
PGPC-evoked calcium spike responses are efficiently reduced by HC-030331 and BCTC, tetrodotoxin, and ω-conotoxin. **(A)** Representative calcium imaging trace of DRG neurons before application of PGPC. **(B–D)** Representative examples of calcium responses of DRG neurons after local application of either PGPC (30 μM, in **B**), AITC (10 μM, in **C**), or capsaicin (1 μM, in **D**). **(E–H)** Calcium responses of PGPC stimulated DRG neurons in presence of inhibitors of TRPA1 and TRPV1 (10 μM HC-030331 plus 1 μM BCTC, in **E**), TTX (500 nM, in **F**) to inhibit TTX-sensitive voltage-gated sodium channels, nifedipine (5 μM, in **G**) to inhibit L-type voltage-gated calcium channels, and ω-CTX (GVIA, 30 nM, in **H**). **(I)** Representative PGPC-induced calcium response after the washout of ω-CTX for 30 min. **(J)** Number of PGPC-induced calcium spikes under indicated conditions. **(K)** Area under curve (AUC) analysis of PGPC-induced calcium responses under indicated conditions. [in **J,K**: PGPC: *n* = 70 cells; PGPC + HC-030331 + BCTC: *n* = 21; PGPC + TTX: *n* = 8; PGPC + nifedipine: *n* = 14; PGPC + ω-CTX (GVIA): *n* = 8. PGPC post-inhibition with ω-CTX: *n* = 8. Cells prepared from 3 mice; mean ± SEM; One-way ANOVA *post-hoc* Holm-Sidak; ^*^p < 0.05, ^**^p < 0.01; ^***^p < 0.001].

To find out whether the calcium spike behavior in response to a PGPC stimulus depends on voltage-gated calcium channels, we stimulated DRG neurons locally with 30 μM PGPC using a drug application system (Figure 3). Before stimulation, cells showed no spontaneous calcium spike activity in absence as well as in presence of various ion channel inhibitors (Figure 3A). Local application of PGPC induced repetitive calcium spike signals with a fast onset and a slowly decaying signal (Figure 3B). In contrast to PGPC, 10 μm AITC induced a typical calcium transient with a slow onset and a slowly decaying signal (Figure 3C), while 1 μM Capsaicin induced a long-lasting calcium transient (Figure 3D). PGPC-induced calcium spikes were strongly inhibited by the TRPA1 and TRPV1 antagonists HC-030031 (10 μM) and BCTC (1 μM) (Figures 3E,J,K). Calcium spike behavior in response to PGPC stimulation was also reduced in presence of TTX (500 nM, Figures 3F,J,K). Notably, while the L-type Ca_V_-channel blocker nifedipine failed to inhibit the PGPC-induced calcium spikes at a concentration of 5 μM (Figures 3G,J,K), ω-conotoxin (CTX, GVIA), a blocker of the high-voltage activated calcium channel Ca_V_2.2 (N-type; Catterall et al., 2005) could almost completely block the PGPC-induced calcium spike activity (Figures 3H,J,K). After a washout period of 30 min, PGPC could again induce the calcium spike behavior in the same neurons (Figures 3I–K). Recovery from CTX inhibition was not complete and the PGPC-stimulus was less efficient (*t*-test PGPC before inhibition vs. PGPC post-inhibition: *p* = 0.013). In summary, this experiment shows that PGPC has the ability to trigger repetitive firing of voltage-dependent calcium spikes in a large portion of DRG neurons.

### PGPC and OxPAPC induce action potential firing of small-diameter neurons

**Figure 4:**
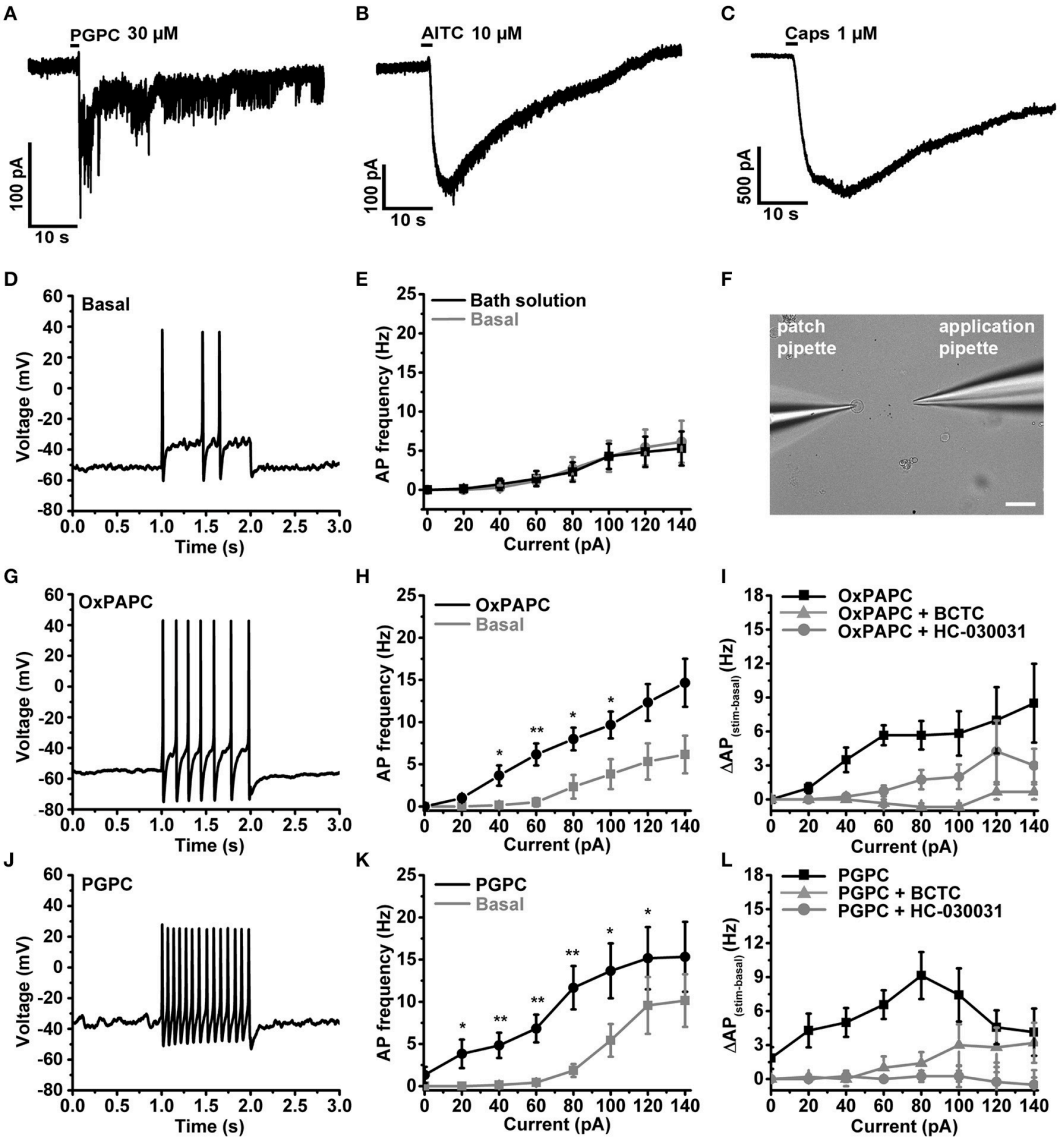
OxPL facilitate action potential firing of DRG neurons. **(A–C)** Representative currents induced by PGPC (30 μM), AITC (10 μM), or Capsaicin (1 μM) stimulation at −60 mV. **(D)** Representative train of action potentials obtained from a murine DRG neuron under control condition (Basal) in response to a current injection of 80 pA for 1 s. **(E)** Action potential frequencies obtained from experiments as shown in **A** as a function of injected current under control conditions (Basal) and during application with external solution (external solution; *n* = 7 from 3 mice). Lines connect data points for clarity. **(F)** Image showing the focal application of solution to individual DRG neurons during current-clamp recordings. **(G)** Representative train of action potentials, recorded from a murine DRG neuron in response to a 1 s current injection of 80 pA after application of 30 μM OxPAPC. **(H)** Action potential frequency obtained from experiments as shown in **D** before (Basal) and during OxPAPC (30 μM) stimulation as a function of injected current (*n* = 6 from 4 mice; paired *t*-test; ^*^p < 0.05; ^**^p < 0.01; *p*-values see Table 1). Lines connect data points. **(I)** Changes of the firing frequency of DRG neurons, ΔAP_(stim−basal)_, induced by stimulation with 30 μM OxPAPC in absence (OxPAPC) or presence of either 1 μM of the TRPV1 inhibitor BCTC (OxPAPC + BCTC) or 10 μM of the TRPA1 inhibitor HC-030031 (10 μM; OxPAPC + HC-030031; *n* = 3–6 from 3 mice; mean ± SEM; One-way ANOVA *post-hoc* Holm-Sidak; *p*-values see Table 2). **(J)** Representative train of action potentials, recorded from a murine DRG neuron in response to a 1 s current injection of 80 pA after stimulation with 30 μM PGPC. **(K)** Action potential frequency obtained from experiments as shown in G before (Basal) and during stimulation with 30 μM PGPC as a function of injected current. (*n* = 7 from 5 mice; mean ± SEM; paired *t*-test; ^*^p < 0.05; ^**^p < 0.01; *p*-values see Table 1). **(L)** Changes of the firing frequency of DRG neurons, ΔAP_(stim−basal)_, induced by application of 30 μM PGPC in absence (PGPC) or presence of either 1 μM of the TRPV1 inhibitor BCTC (OxPAPC + BCTC) or 10 μM of the TRPA1 inhibitor HC-030031 (OxPAPC + HC-030031; *n* = 4–7 from 3 mice; mean ± SEM; One-way ANOVA *post-hoc* Holm-Sidak; *p*-values see Table 2). For **I,L**: For overview purposes, significance marks can be found in Table 2.

We recently showed that PGPC induces long-lasting inward currents in HEK293 cells stably expressing TRPA1 (Oehler et al., 2017). To test whether long-lasting PGPC-induced inward currents are also present in small-diameter DRG neurons, we performed voltage-clamp experiments with these neurons. Similar to our previous results obtained with transfected HEK293 cells (Oehler et al., 2017), application of PGPC triggered a small, yet long-lasting inward current response in the neurons. Local application of AITC (Figure 4B) or capsaicin (Figure 4C) for 500 ms resulted in typical corresponding TRP ion channel responses. Next, whole-cell current-clamp recordings were performed to investigate the influence of OxPL on the action potential (AP) firing characteristics of DRG neurons. As shown in Figure 4, trains of action potentials were recorded from individual DRG neurons before and after focal and acute application of OxPL. Small-diameter DRG neurons (21.83 ± 1.78 pF; mean ± SEM; *n* = 32 cells) were locally stimulated with OxPAPC and PGPC for 500 ms with the help of a wide-opened patch clamp pipette and a computer controlled pressure ejection system. The current injection-dependent AP frequency was determined before and after acute application of PGPC. Application of vehicle (extracellular solution) alone did not increase the rate of AP-firing, confirming that the application procedure is appropriate for these experiments (Figures 4D,E). OxPAPC (Figures 4G–I), and PGPC (Figures 4J–L) induced a current-dependent increase in the AP frequency, which could be reduced by the TRPV1 inhibitor BCTC and the TRPA1 inhibitor HC-030031.

Since PGPC acts via TRPA1 and TRPV1 in DRG neurons, we asked how the PGPC-induced receptor potentials are integrated to induce action potential firing. As demonstrated earlier, Na_V_1.9 is the best candidate to serve as a subthreshold amplifier of receptor potentials (Cummins et al., 1999; Herzog et al., 2001). Furthermore, Na_V_1.9 triggers the spontaneous calcium-spike behavior of embryonic motoneurons (Subramanian et al., 2012). Therefore, we asked whether Na_V_1.9 ion channels drive the AP firing and calcium spike response of small-diameter neurons upon an OxPL stimulus.

### Minor contribution of Na_V_1.9 on OxPL-induced AP firing under steady-state conditions

**Figure 5:**
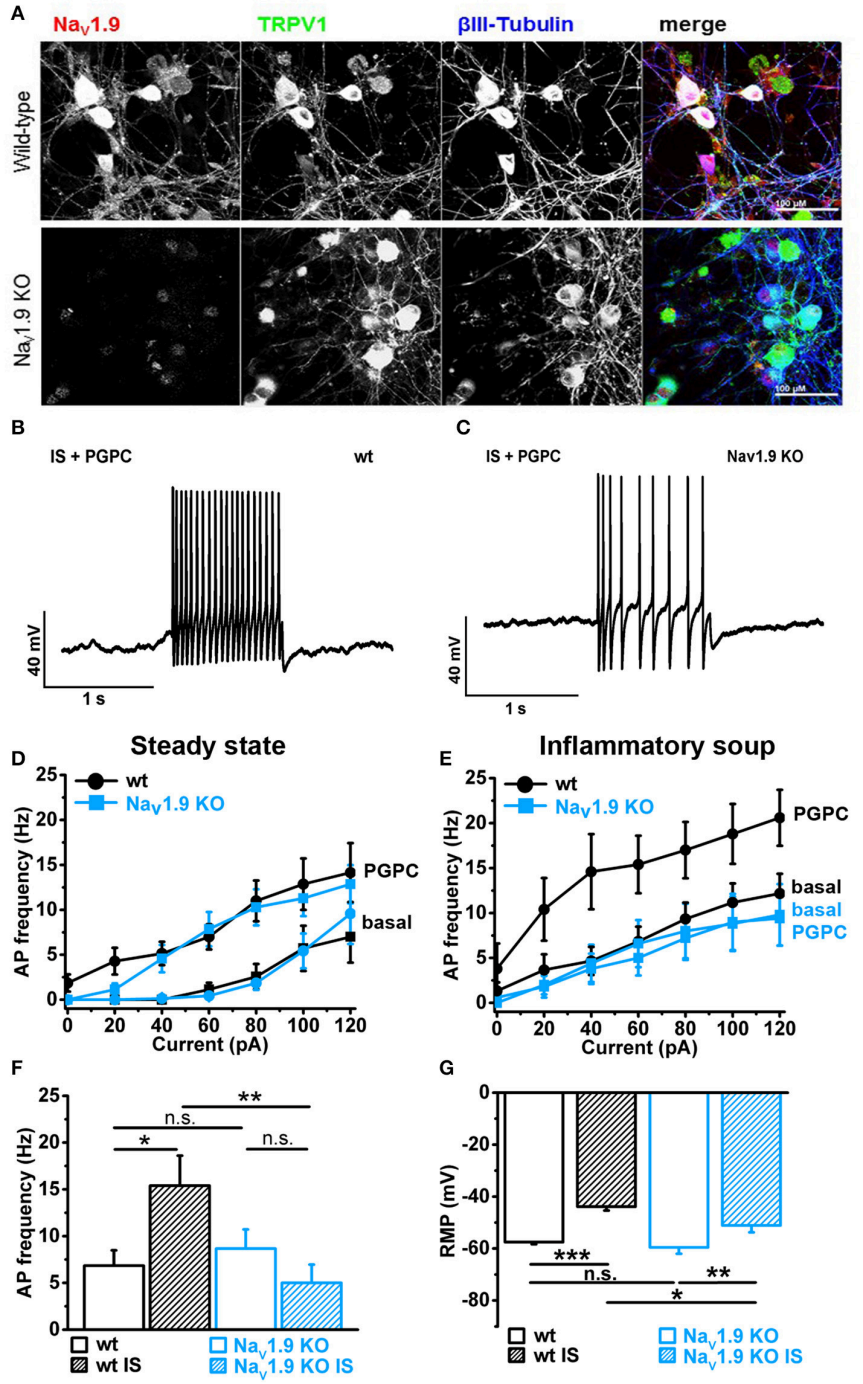
Inflammatory mediators recruit Na_V_1.9 to potentiate OxPL function. **(A)** Indirect immunofluorescent staining of 48 h old primary DRG cultures from Bl6 wt mice or Na_V_1.9 KO mice were stained against Na_V_1.9 (red), TRPV1 (green) and βIII-Tubulin (blue). **(B)** Representative train of action potentials, recorded from a murine DRG neuron in response to a 1 s current injection of 80 pA after incubation in IS for 30 min and application of 30 μM PGPC. **(C)** Representative train of action potentials from a murine Na_V_1.9 KO DRG neuron in response to a 1 s current injection of 80 pA after incubation in IS for 30 min and application of 30 μM PGPC. **(D)** Frequency of action potential firing of wild-type and Na_V_1.9 KO neurons in response to variable current injections ranging from 0 to120 pA before (Basal) and after stimulation with 30 μM PGPC under steady state conditions (mean ± SEM; *n* = 7 from 6 mice; statistics see Table 3). **(E)** Action potential frequencies of wild-type and Na_V_1.9 KO DRG in response to variable current injections before (Basal) and after application of 30 μM PGPC and pretreatment with inflammatory soup [*n* = 5 from 5 mice (wild-type); *n* = 5 from 3 mice (Na_V_1.9 KO); mean ± SEM; statistics see Table 3]. **(F)** Frequency of action potentials obtained after PGPC application during 60 pA current injections for 1 s from wild-type and Na_V_1.9 KO DRG neurons without (open bars) or with (striped bars) pretreatment of cells with inflammatory soup (IS) [*n* = 5–7 of 3 mice; mean ± SEM; one-way ANOVA; *post-hoc* Holm-Sidak; *F*_(3, 21)_ = 3.86; ^*^p = 0.014; ^**^p = 0.0054]. **(G)** Resting membrane potential (RMP) of wild-type and Na_V_1.9 KO DRG neurons obtained under either steady state conditions (wt: *n* = 19; Na_V_1.9 KO: *n* = 12) or after pretreatment with inflammatory soup [wt: *n* = 8; Na_V_1.9 KO *n* = 9; mean ± SEM; one-way ANOVA; *post-hoc* Holm-Sidak; *F*_(3, 46)_ = 13.34; ^*^p < 0.019; ^**^p = 0.0029; ^***^p = 3.95E-06].

First, we verified that Na_V_1.9 is expressed in our primary DRG cultures by labeling these cells with an antibody against the C-terminus of mouse Na_V_1.9 (Subramanian et al., 2012). Counterstains against TRPV1 and the neuronal marker βIII-Tubulin served as control. Anti-Na_V_1.9 immunoreactivity was observed in large and small-diameter primary DRG neurons (Figure 5A, upper panel). This immunoreactivity was almost lost in DRG cultures obtained from Na_V_1.9 KO mice, albeit a certain background label of nuclei was routinely observed (Figure 5A, lower panel).

Electrophysiological analysis of these neurons revealed that the PGPC-induced increase in stimulated action potential firing was almost indistinguishable between Na_V_1.9 KO neurons and wild-type neurons (Figures 5B–D; wild-type data taken from Figure 4K).

### Inflammatory mediators recruit Na_V_1.9 to potentiate OxPL function

In DRG neurons, a persistent sodium current can be increased by intracellular GTP or by non-hydrolysable GTP analogs (Baker et al., 2003). This GTP up-regulated persistent sodium current requires Na_V_1.9 (Östman et al., 2008). Furthermore, a soup of exogenously applied inflammatory mediators can rapidly potentiate Na_V_1.9 channel activity to increase AP firing in response to appropriate electrical stimulation (Maingret et al., 2008). These studies suggested that Na_V_1.9 may drive action potential firing under inflammatory conditions.

To address whether AP firing to an OxPL stimulus is potentiated by inflammatory mediators, we applied PGPC to DRG neurons before and after pretreating the cells for 30 min with an inflammatory soup (IS) containing 5 nM bradykinin, 100 nM histamine, 50 nM PGE_2_. In this case, the AP frequency to a PGPC stimulus was substantially increased in comparison to the non-treated control (Figures 5B,E). This potentiating effect was fully lost in Na_V_1.9 KO small-diameter neurons (Figures 5C,E,F). To sum up, sensitized wild-type neurons respond to PGPC application with increased action potential firing and the potentiation of neuronal activity in response to PGPC-TRP activation is driven by Na_V_1.9. The resting membrane potential (RMP) of wild-type and Na_V_1.9 KO neurons was not different under steady-state conditions (Figure 5G). The inflammatory soup (IS in Figure 5G), however, caused an increased RMP in both wt and Na_V_1.9 KO neurons, albeit this effect was much stronger in the wt DRG neurons (Figure 5G). Together, these results provide evidence that Na_V_1.9 links the signaling of an endogenous nociceptive chemical activator of TRP ion channels with an increased responsiveness induced by inflammatory mediators.

**Figure 6:**
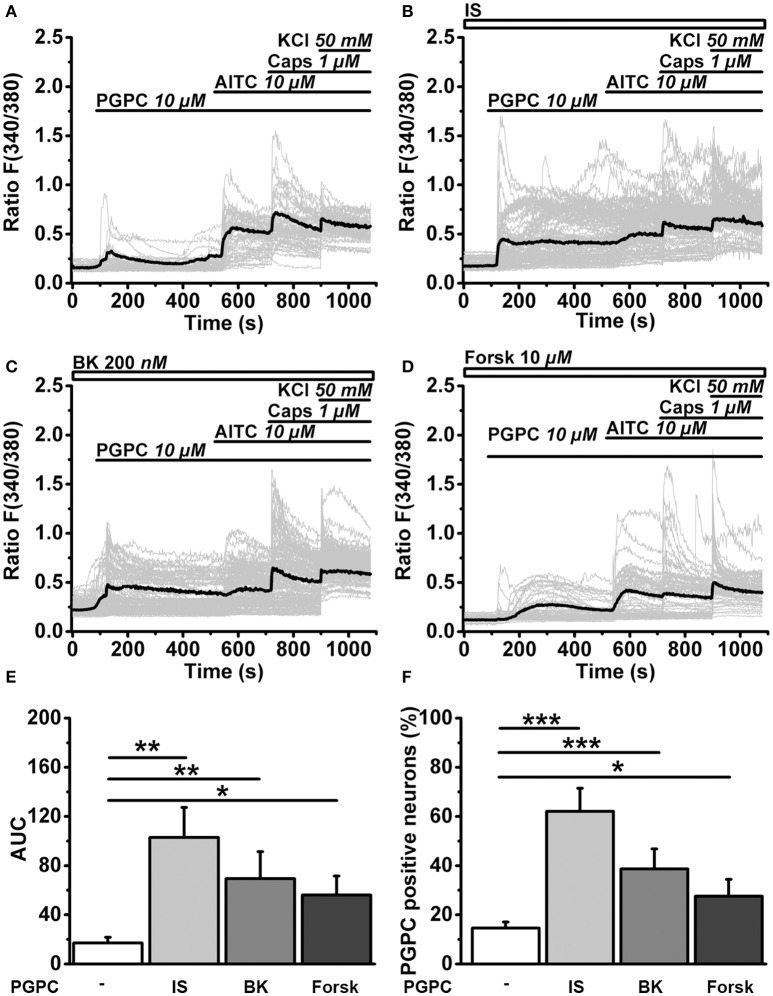
Inflammatory mediators promote OxPL-induced activation. **(A)** Relative changes in (Ca^2+^)_i_ in wild-type DRG neurons loaded with Fura-2-AM, expressed as florescence ratio *F*_(340/380)_ as a function of time. Application of agonists PGPC (10 μM), AITC (10 μM), capsaicin (Caps, 1 μM), and KCl (50 mM) are indicated with horizontal bars. Gray traces are calcium responses of 50 individual cells; the black trace represents the mean response of al experiments. **(B–D)** Identical experiments as shown in A, but after preincubation of cells with either Inflammatory soup (IS, **B**), Bradykinin (BK, **C**), Forskolin (Forsk, **D**). **(E)** Integrated calcium responses from experiments shown in **(A–D)** [*n* = 6–8 from 3 mice; mean ± SEM; One-way ANOVA *post-hoc* Holm-Sidak; *F*_(3, 26)_ = 3.84; ^*^p = 0.045; ^**^p = 0.0029]. **(F)** The percentage of neurons reacting to PGPC stimulation following treatment with inflammatory mediators [*n* = 6–8 from 3 mice; mean ± SEM; One-way ANOVA *post-hoc* Holm-Sidak; *F*_(3, 26)_ = 15.45; ^*^p < 0.05; ^***^p < 0.001].

To monitor the effect of inflammatory mediators on the calcium responsiveness to PGPC, we extended the calcium imaging experiments (Figure 6). In slow ratiometric calcium imaging experiments, PGPC stimulation substantially increased the influx of calcium ions after treatment with inflammatory soup compared to PGPC alone (Figure 6A vs. Figures 6B,E,F). Bradykinin and Forskolin alone were not as effective as the inflammatory soup (Figures 6C–F). The apparent increase in calcium responsiveness to a PGPC stimulus was obvious in two parameters, the area under curve analysis and the percentage of reacting neurons (Figures 6E,F). This experiment confirmed that our inflammatory soup conditions are able to enhance the PGPC stimulus.

### Na_V_1.9 drives calcium spikes

**Figure 7:**
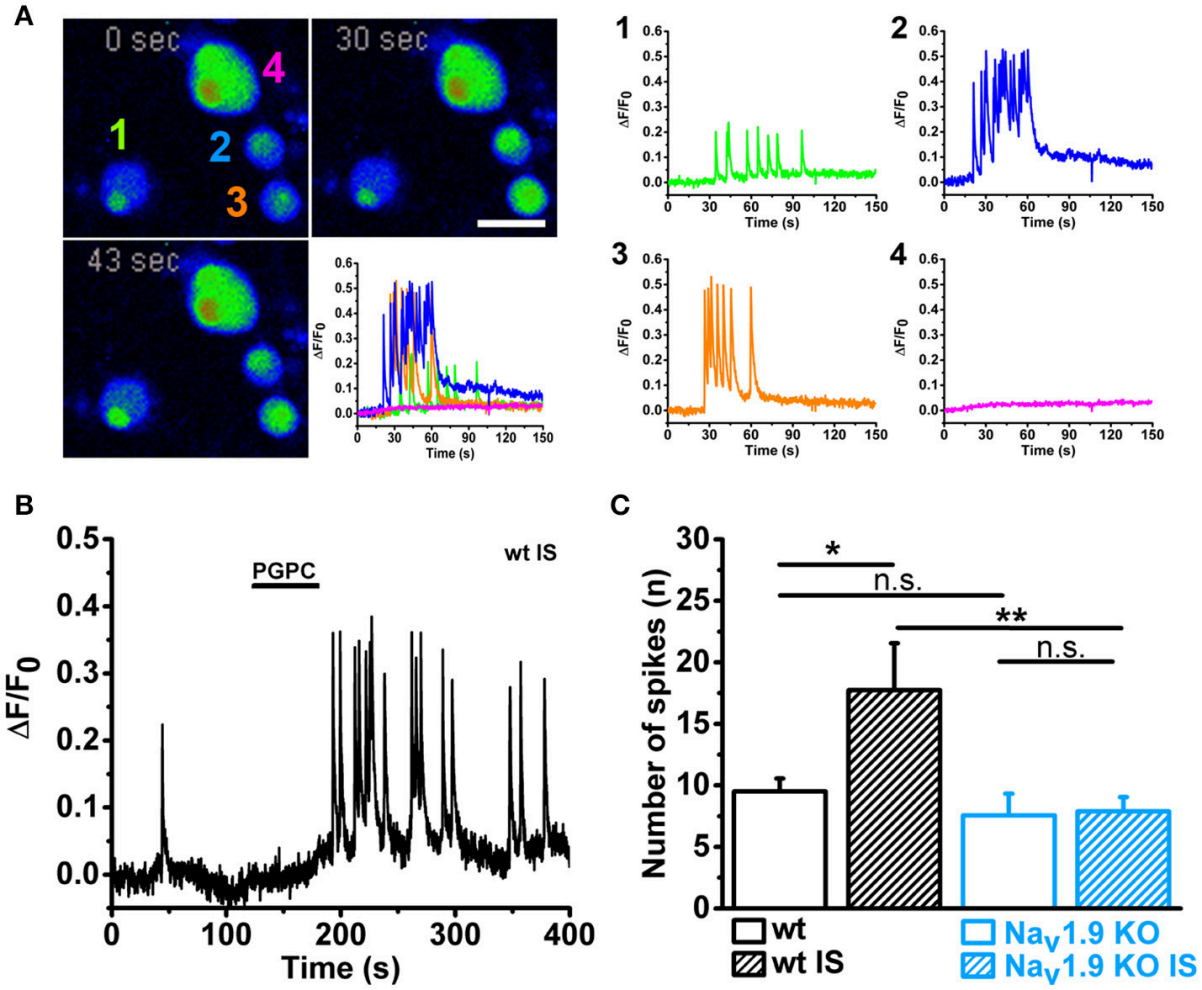
Inflammatory mediators enhance PGPC-induced and Na_V_1.9 dependent calcium spikes in DRG neurons. **(A)** *Left* Time series of pseudo colored images showing DRG neurons loaded with calcium indicator. Calcium evoked spiking of DRG neurons after preincubation with inflammatory mediators and stimulation with PGPC (10 μM; 30 s, 43 s) are displayed in comparison to the basal situation (0 s). The reaction of the four cells imaged in the experiment is shown on the lower right (scale bar = 50 μm). *Right* Calcium traces of the cells shown on the left upon stimulation with PGPC monitored on the left. **(B)** Spiking behavior of a representative wild-type DRG neuron upon stimulation with PGPC following a pretreatment with inflammatory soup. **(C)** Number of PGPC-triggered calcium spikes of wild-type DRG neurons without (open bars; wt *n* = 37 from 5 mice; Na_V_1.9 KO *n* = 9 from 5 mice) or after pretreatment with inflammatory soup [wt *n* = 20; Na_V_1.9 KO *n* = 25; *n* = 9–37 from 5 mice; mean ± SEM; Two-way-ANOVA, Holm-Sidak; *F*_(3, 90)_ = 4.80; ^**^p = 0.0028; ^***^p = 9.64E-04].

Finally, we asked whether the calcium-spike behavior of small-diameter neurons is Na_V_1.9-dependent. Na_V_1.9 function is normally investigated with help of patch clamp recording and specific adaptations in the intracellular solution (for instance internal fluoride) and extracellular milieu (for instance high concentration of tetrodotoxin) are needed to efficiently define the contribution of Na_V_1.9 to neuronal excitation (Baker et al., 2003; Rugiero et al., 2003; Maingret et al., 2008; Östman et al., 2008). On the other hand, Na_V_1.9 has been shown to trigger cell-autonomous calcium spikes in growth cones and somata of primary motoneurons (Subramanian et al., 2012; Wetzel et al., 2013). These data are compatible with the hypothesis that the persistent sodium current by Na_V_1.9 can drive a local excitation cascade, which results in calcium spikes by voltage-activated calcium channels (Wetzel et al., 2013). Theoretically, when a local pulse of PGPC induces a receptor potential, then the persistent sodium current by Na_V_1.9 should be able to drive repetitive calcium spikes. To test this hypothesis, we measured PGPC-induced calcium signals with fast calcium imaging and compared wildtype and Na_V_1.9 KO DRG neurons with or without treatment with inflammatory mediators. As shown in Figure 7, wildtype small-diameter neurons responded with an increased number of typical calcium spikes upon treatment with inflammatory mediators (representative in Figures 7A,B, Supplementary Video 2; response variability of single cells is indicated). The calcium spike response was typically seen in small diameter neurons, and not in larger neurons, as indicated in Figure 7A. In contrast, the inflammatory-mediator dependent increase in calcium spike frequency was fully lost in Na_V_1.9 KO neurons (Figure 7C). Spike behavior correlated with the acute PGPC stimulus and ended after some seconds. Thus, the PGPC stimulus was needed to start the spike behavior. Pretreatment with inflammatory mediators alone was not sufficient to drive a long-lasting or permanent spike behavior of the small-diameter neurons.

**Figure 8:**
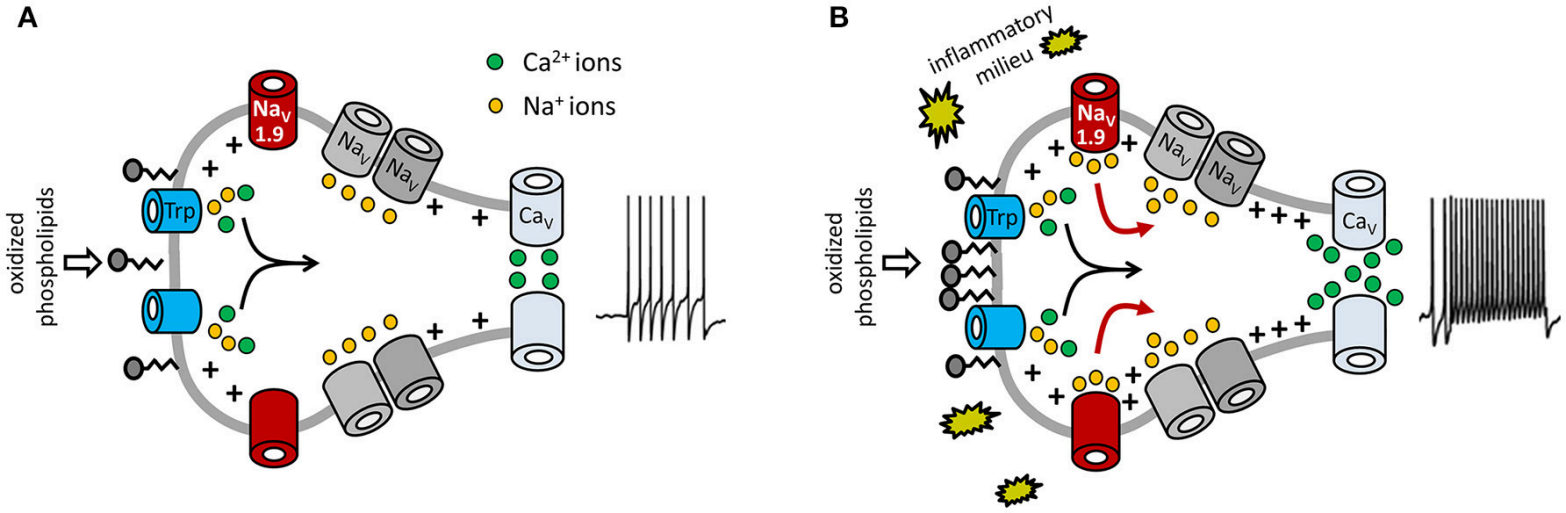
Model for potentiation of OxPL-induced TRP activation by Na_V_1.9. Shown are molecular determinants involved in OxPL-induced excitation of DRG neurons. Black arrows indicate the contribution of TRP-mediated non-selective inward currents in nociceptor excitation. Red arrows indicate the role of Na_V_1.9-dependent sodium currents to an increased excitability of nociceptive neurons under inflammatory conditions. **(A)** Under steady-state conditions, a certain amount of oxidized phospholipids is produced and can stimulate nociceptors via TRPA1 and TRPV1. However, in this situation, acute activation of TRP ion channels is moderate. PGPC is known to integrate into biological membranes, indicating that PGPC might activate TRP channels indirectly, as discussed recently (Oehler et al., 2017). **(B)** Inflammatory mediators sensitize the peripheral nerve endings and increase Na_V_1.9 function. This subthreshold active sodium channel mediates a persistent sodium current that increases the potency of the cell to fire action potentials and to answer with repetitive calcium spikes. In this model, Na_V_1.9 behaves like a switch to integrate chemical irritant stimuli under inflammatory conditions. Besides OxPL such as PGPC, other endogenous proalgesic metabolites might use the here indicated switch-behavior of Na_V_1.9 to potentiate their receptor-mediated signals under inflammatory conditions.

In summary, our data imply that Na_V_1.9 behaves like a switch to potentiate OxPL-induced receptor potentials, specifically under inflammatory conditions. The cells then respond with an increased action potential firing frequency and also with an increased number of calcium spikes (a signaling model is shown in Figure 8).

## Discussion

In a recent study we showed that OxPL are generated in inflamed paw tissue, are proalgesic intrinsic irritants and mediate their function via TRPA1 and TRPV1 ion channels (Oehler et al., 2017). Here, we asked how the rather brief activation of TRP ion channels by OxPL affects the activity of small-diameter DRG neurons, a cellular model for nociceptors. Using calcium imaging techniques and electrophysiological methods, we show that OxPL-triggered activity of small-diameter DRG neurons is potentiated after pretreatment with inflammatory mediators. This signaling pathway involves the voltage gated sodium channel Na_V_1.9 as an essential and non-redundant mediator.

### Oxidized phospholipids: endogenous excitants of DRG neurons

OxPLs are highly reactive, transient endogenous irritants of nociceptors (Liu et al., 2016; Oehler et al., 2017). During inflammation, immune cells produce more reactive oxygen species (ROS; Catala, 2009), which can oxidize phospholipids of cellular membranes leading to the formation of OxPL (Freigang, 2016). OxPLs have been shown to activate several receptors present on nociceptive neurons: TRPA1, TRPV1, and TRPC5 (Al-Shawaf et al., 2010; Liu et al., 2016; Oehler et al., 2017). So far, the mechanism of action of OxPL remains elusive and a “one-for-all” mechanism of OxPL action does not seem to exist (Oehler et al., 2017). For instance, the activation of TRPA1 by OxPL via an interaction with cysteines in the TRP N-terminus requires an electrophilic substance, such as the mustard oil compound AITC (Hinman et al., 2006; Macpherson et al., 2007) or electrophilic OxPAPC compounds (Oehler et al., 2017). However, since PGPC is a stable but non-electrophilic OxPL, it appears plausible that OxPL might activate TRP channels indirectly. There is evidence, that PGPC integrates into the lipid bilayer of plasma membranes and modulates functional properties of lipids and proteins (Stemmer and Hermetter, 2012) so that finally rearrangements of the plasma membrane might induce a mechanical activation of TRPA1 and even TRPV1 (Kwan et al., 2006; Jansson et al., 2013; Saghy et al., 2015). A similar mode of action has been proposed for bacterial lipopolysaccharides (Meseguer et al., 2014). Here, we revealed that PGPC activates a TRPA1 mutant lacking the characteristic binding site for electrophile agonists of TRPA1 (Macpherson et al., 2007). In contrast to the high potency of typical exogenous TRPA1 and TRPV1 stimuli, such as AITC or capsaicin, PGPC-induced currents on small DRG neurons are rather tiny (Oehler et al., 2017).

OxPL are continuously present and produced in subcutaneous tissue (Oehler et al., 2017). Because OxPLs are abundant in tissue and are produced at higher levels during inflammation, it appears plausible that a mechanism is needed to distinguish between healthy steady-state conditions or inflammatory periods. We propose that Na_V_1.9 is an essential and non-redundant part of a switch mechanism that helps to distinguish the non-inflamed resting state from the diseased state, so that OxPL stimuli on TRP channels are more potent under inflamed conditions, but not under steady-state, healthy conditions. Furthermore, inflammatory condition might increase the Na_V_1.9 expression rate and protein abundance (Bennett and Woods, 2014; Fischer et al., 2017). However, Na_V_1.9 mRNA expression and immunoreactivity is already high under steady-state conditions (Subramanian et al., 2012; Figure 4). This argues against the idea that the main effect of inflammatory mediators is the stimulation of *de novo* Na_V_1.9 expression. An important feature of Na_V_1.9 is its ability to generate persistent sodium currents (Cummins et al., 1999; Herzog et al., 2001). Therefore, it is likely that this current drives the observed calcium spike behavior of the neurons. The function of Na_V_1.9 to support long-lasting calcium spikes was also observed in motoneurons, where it is a cell-autonomous and spontaneous phenomenon (Subramanian et al., 2012; Wetzel et al., 2013). In motoneurons, Na_V_1.9 triggered calcium spikes stimulate axonal elongation (Subramanian et al., 2012; Wetzel et al., 2013). In small-diameter neurons, the increased calcium spike response might support axonal growth effects such as axonal sprouting.

### Role of Na_V_1.9 as an excitability mediator

In adult small DRG neurons, several voltage-gated sodium channels (VGSC) are expressed controlling nociceptor excitability and action potential firing (Bennett and Woods, 2014; Waxman and Zamponi, 2014). In particular, Na_V_1.7, Na_V_1.8 and Na_V_1.9 are of physiological relevance in small-diameter sensory neurons, and all three subtypes have been linked to human pain disorders (for review: Dib-Hajj et al., 2010; Bennett and Woods, 2014; Habib et al., 2015). Na_V_1.7 and Na_V_1.8 participate in the fast upstroke of action potentials, while Na_V_1.9 influences cellular excitability close to the resting membrane potential (Cummins et al., 1999; Priest et al., 2005; Maingret et al., 2008; Östman et al., 2008; Dib-Hajj et al., 2010; Leipold et al., 2013; Habib et al., 2015). The discovery of rare mutations in the gene encoding for Na_V_1.9 in individuals suffering from either a complete loss of pain perception, painful peripheral neuropathy or episodic pain syndromes highlighted Na_V_1.9 as a potential target to treat pain (Leipold et al., 2013; Zhang et al., 2013; Huang et al., 2014, 2017; Woods et al., 2014; Han et al., 2015). However, one should consider that Na_V_1.9 is not only functionally important in nociceptors, but serves as an excitability mediator in many types of neurons. For instance in embryonic motoneurons the activity of Na_V_1.9 triggers cell-autonomous calcium spikes in growth cones to support activity-dependent axon elongation (Subramanian et al., 2012; Wetzel et al., 2013). Notably, these Na_V_1.9-triggered calcium spikes are reduced in preclinical models mimicking spinal muscular atrophy (Jablonka et al., 2007; Wetzel et al., 2013). A broader function of Na_V_1.9 is also supported by the complex phenotype observed in individuals carrying the L811P mutation (Leipold et al., 2013; Woods et al., 2014). Besides the loss of pain perception, clinical signs include severe gastrointestinal symptoms such as intestinal dysmotility, which is in accordance with Na_V_1.9 function in myenteric sensory neurons of rodents (Rugiero et al., 2003). Furthermore, muscular weakness and signs of delayed motor development are in line with a function in the development or maintenance of the motor system (Subramanian et al., 2012; Leipold et al., 2013; Woods et al., 2014). Therefore, a deeper knowledge of the activation cascades upstream of Na_V_1.9 is needed before systemically applied therapeutics targeting Na_V_1.9 can become safe.

### Inflammatory mediators and Na_V_1.9 function

*In vitro* and *in vivo* data from animal studies support a role for Na_V_1.9 in inflammatory pain (Baker et al., 2003; Priest et al., 2005; Amaya et al., 2006; Maingret et al., 2008; Östman et al., 2008). It has been shown that inflammatory mediators, when applied as a mixture similar to inflammatory milieu (inflammatory soup), are able to stimulate the Na_V_1.9 current, to amplify subthreshold electrical stimuli and to increase action potential firing in response to depolarizing inward currents (Maingret et al., 2008). Sensitization of DRG neurons by inflammatory mediators can occur by different mechanisms and several have been proposed to explain the increased action potential firing rate induced by inflammatory mediators (Momin and Mcnaughton, 2009). In accordance with a study by Maingret et al. (2008), we observed that the RMP was not significantly different between small wild-type and Na_V_1.9^−/−^ DRG neurons. However, after treatment of DRG neurons with inflammatory soup, we observed a slightly more negative resting membrane potential (RMP) in Na_V_1.9 KO neurons than in wild-type control neurons (see Figure 5E). This suggests that Na_V_1.9 contributes to an increased RMP under inflammatory conditions and therefore increases the ease with which action potentials are elicited after OxPL stimulation. Thus, a significant fraction of the Na_V_1.9 channels seem to be active after treatment with inflammatory mediators for 30 min.

Inflammatory mediators can stimulate or sensitize terminals of nociceptors through diverse signaling pathways (Basbaum et al., 2009; Ji et al., 2014, 2016; Denk et al., 2017). Inflammatory mediators include classic mediators (for example, bradykinin, prostaglandins, protons, ATP and the neurotrophin nerve growth factor, NGF), but also transmitters and neuromodulatory substances such as substance P, calcitonin gene-related peptide or the neurotrophin brain-derived neurotrophic factor (BDNF). Not much is known about signaling cascades leading to an activation of Na_V_1.9. When inflammatory mediators were applied conjointly to DRG neurons, the concerted action of the inflammatory soup was needed to rapidly potentiate Na_V_1.9 currents (Maingret et al., 2008). We applied the inflammatory soup for about 30 min, before we performed the OxPL stimulation. To find out the causal link between inflammatory mediator action and Na_V_1.9 activation is challenging. A molecular target side in Na_V_1.9 (e.g., a specific channel-activating phosphorylation site) is not yet known for this sodium channel. Molecular reconstitution of the signaling pathways leading to increased Na_V_1.9 activity would be needed. Indeed, after almost one decade of low progress, important improvements have been achieved in the last years and new heterologous Na_V_1.9 expression tools were developed (Subramanian et al., 2012; Leipold et al., 2013; Vanoye et al., 2013; Lin et al., 2016). However, a better biochemical access to functional Na_V_1.9 protein is still needed before it will be possible to causally link Na_V_1.9 activation with specific signaling pathways. Anyhow, we assume that signaling cascades downstream of the neurotrophin receptors for NGF or BDNF are plausible candidates here. These signaling cascades are powerful enough to drive long-lasting excitability effects, translational changes, and growth effects, as already described for many types of neurons (Blum et al., 2002; Subramanian et al., 2012; Wetzel et al., 2013; Sasi et al., 2017). The role of inflammatory mediators might be indirect, by first establishing a higher spontaneous activity to sensory neurons, which would then drive the activity-dependent regulation of neurotrophins. Therefore, it will become a challenging endeavor to identify the possible molecular mechanism by which Na_V_1.9 can be activated through the complex interplay between inflammatory mediators and neurotrophins.

## Conclusion

In conclusion, the present study demonstrates that an inflammatory milieu recruits Na_V_1.9 to potentiate OxPL function via TRP ion channels. The data suggest that Na_V_1.9 serves as master switch distinguishing between resting excitability and increased excitability under inflammatory conditions.

## Author contributions

RB, BO, EL, and HR designed research, CM, CS, MM, JH, and RB performed research, CM and CS analyzed data, CM, CS, EL, and RB wrote paper.

### Conflict of interest statement

The authors declare that the research was conducted in the absence of any commercial or financial relationships that could be construed as a potential conflict of interest.

## Supplementary material

Supplementary Figure 1 Cells positive for the glial cell marker GFAP (glial fibrillary acidic protein) are a second abundant cell type in adult prepared DRG neuron cultures. Cultures were labeled at DIV3 with an antibody against GFAP (Acris, DP014, 1/2000) and with the neuronal marker Tuj1 (Acris, MO15013, 1/2000). DAPI was used to label cell nuclei.

Supplementary Video 1 Calcium imaging movie. Primary DRG neurons were stimulated with PGPC, AITC, Capsaicin and KCl. The indicated cell (A) shows spike-like calcium responses to PGPC and during a subsequent AITC stimulus.

Supplementary Video 2 Calcium imaging movie. Primary DRG neurons were pretreated with inflammatory soup and then stimulated with an acute PGPC stimulus. Three neurons with small diameter respond with calcium spikes, while one neuron remains non-responsive to PGPC. The spike behavior ends after some seconds (~40 s).
