# Supplementary figures and images for: NaV1.9 Potentiates Oxidized Phospholipid-Induced TRP Responses Only under Inflammatory Conditions
Source: Front Mol Neurosci. 2018 Jan 23;11:7. doi: 10.3389/fnmol.2018.00007 (PMC5787077; doi:10.3389/fnmol.2018.00007)

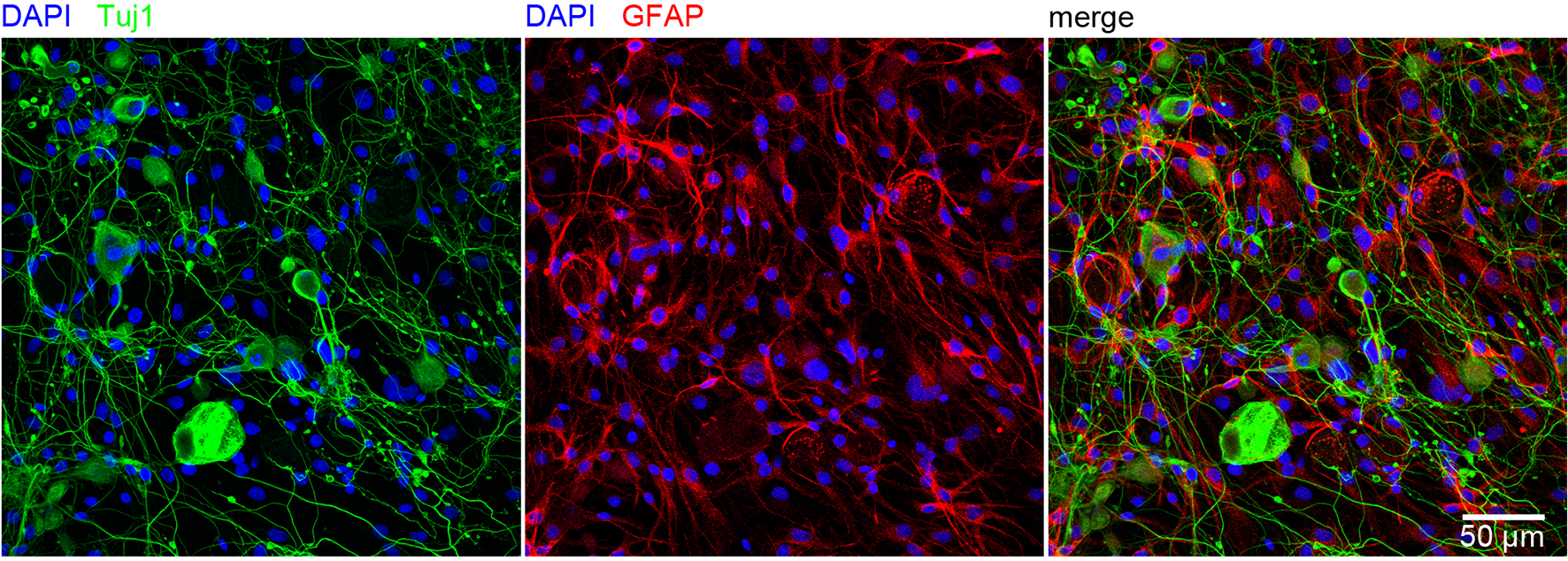

Supplement: Supplementary Figure 1 — Cells positive for the glial cell marker GFAP (glial fibrillary acidic protein) are a second abundant cell type in adult prepared DRG neuron cultures. Cultures were labeled at DIV3 with an antibody against GFAP (Acris, DP014, 1/2000) and with the neuronal marker Tuj1 (Acris, MO15013, 1/2000). DAPI was used to label cell nuclei. [file Image1.TIF]
